# Impact of Light/Dark Cycle Patterns on Oxidative Stress in an Adriamycin-Induced Nephropathy Model in Rats

**DOI:** 10.1371/journal.pone.0097713

**Published:** 2014-05-22

**Authors:** Begoña M. Escribano, Antonia Moreno, Inmaculada Tasset, Isaac Túnez

**Affiliations:** 1 Department of Cell Biology, Physiology and Immunology, Faculty of Veterinary Medicine, Córdoba University, Córdoba, Spain; 2 Department of Biochemistry and Molecular Biology, Faculty of Medicine, Maimónides Institute of Biomedical Research of Córdoba (IMIBIC), Córdoba University, Córdoba, Spain; The University of Manchester, United Kingdom

## Abstract

The principal goal of this study was to determine the effect of the photoperiod on oxidative damage biomarkers in rats submitted to different light/darkness patterns, in a hyperlipidemic nephropathy model (induced by adriamycin), as well as its possible relationship with melatonin and leptin secretion rhythms. To test this hypothesis, six different groups were used (N = 6 rats per group): control (12 h/12h light:dark); exposure to permanent illumination (24 h light); exposure to darkness (22 h dark); injected with adriamycin, 12h/12h light:dark; injected with adriamycin + exposure to permanent illumination and injected with adriamycin + exposure to darkness (22 h dark). The different photoperiods were begun two weeks prior to medication and were maintained up to the day of the animal's sacrifice, ten days after medication. The following parameters were analysed: i) weight evolution; ii) in plasma: urea, creatinine, uric acid, total proteins, albumen, lactate dehydrogenase, creatinine-quinase, aspartate aminotransferase, alanine aminotransferase and total cholesterol; iii) in urine: urea, creatinine, total proteins and microalbumen; iv) biomarkers of oxidative damage in kidneys, heart, liver and brain: lipoperoxides, total glutathione, reduced glutathione, catalase, glutathione peroxidase, glutathione reductase and glutathione transferase; v) melatonin (pineal gland tissue and plasma) and leptin (plasma). From the results obtained it was concluded that the administration of adriamycin generated oxidative stress in renal, cerebral, hepatic and cardiac tissue. Additionally, in the healthy animal, but of a lesser relevance in the adriamycin animal, permanent light worsened the oxidative stress, whereas darkness improved it. This could be related to the circadian rhythm of the inverse release shown by melatonin and leptin, accentuating the release of melatonin in the darkness phase and that of leptin in the light phase. The correlation between melatonin and leptin in the healthy animal seemed to confirm the relationship between both variables and their influence on oxidative damage biomarkers.

## Introduction

Light is a powerful element in circadian, neuroendocrine and neurobehavioural regulation and it has a profound influence on the health and wellbeing of all mammals, including laboratory animals [1]. Many hormones and enzymes are fundamental to life, and these are secreted following a circadian pattern in accordance with the photoperiod. [2]–[4].

In vertebrates, melatonin is secreted during darkness as a hormonal message of the photoperiod [5]. The circadian release of melatonin is regulated by an oscillator, which is situated in the hypothalamic suprachiasmatic nucleus (SCN) [6]–[7]. This oscillator is usually entrained to a 24-h rhythm [8]–[9] by environmental lighting conditions, which are perceived in the retina by rods, cones and intrinsically photosensitive retinal ganglion cells [10]. Circulating levels of melatonin are high at night and low in the daytime [11].

Leptin plasma concentration, secreted by adipose cells, increases during the afternoon and reaches its peak between midnight and approximately 2:30 a.m. [12], with considerable interindividual variations [13].

Despite the different circadian secretion patterns of leptin and melatonin, both hormones seem to be related although these results are controversial ones [14]–[16].

Interestingly, daily rhythms of some enzymes related to oxidative stress such as superoxide dismutase (SOD), glutathione peroxydase (GPx), and glutathione reductase (GRd) activities, as well as glutathione and malondialdehyde levels, have been demonstrated as being in the brain [17]–[18]. An excess in the production of free radicals has been involved in a large number of diseases.

Melatonin and leptin have been related to oxidative stress, and both hormones might be exceedingly important in the regulation of certain diseases. Thus, melatonin seems to protect against oxidative stress [19]–[25] and leptin increases the generation of reactive oxygen species (ROS) [26]–[27].

The principal goal of this study was to determine the effect of the photoperiod on oxidative stress in a hyperlipidemic nephropathy model in wistar rats. To test this hypothesis, different photoperiod patterns were used. The hyperlipidemic nephropathy in rats was induced by adriamycin. Adriamycin (AD), commonly used as an antitumoral antibiotic in humans. It provides an ideal model of oxidative stress in rats accompanied by a type of hyperlipidemic nephropathy which is reasonably similar to human focal glomerulesclerosis from a histological point of view [28].

## Materials and Methods

### Animals

All the animal care and research proceedings in this study were carried out in accordance with the Directives 86/609/EEC and 2010/63/EU, the R.Ds 223/1988, 1201/2005 and 53/2013 and the Law 32/2007 of the European Community. They have also been approved by Córdoba University's Committee of Bioethics.

The study was performed on 36 male Wistar rats (250–300 g in weight) aged 3 months (Charles Rivers; Barcelona, Spain). At the beginning of the study all the animals were submitted to controlled temperature conditions (22+/−2°C) and light (12 h. light/12 h darkness, L-D). They had access to water and food *ad libitum* (Purina, Barcelona).

### Treatments

The animals were grouped randomly in sixes, as follows:

  1. Control (12 h/12h L:D)
  2. Exposure to permanent illumination (24 h L)
  3. Exposure to darkness (22 h D) with sessions of light between 4 and 6 p.m.
  4. Injected with adriamycin (AD), 12h/12h L:D
  5. AD + L
  6. AD + D

The different photoperiods were begun two weeks prior to medication and were maintained up to the day of the animal's sacrifice,ten days after medication.

The AD (doxorubicin chlorhydrate. Farmiblastin, from Pharmacia and Upjohn) was administered by intraperitoneal injection on two consecutive days (7.5 mg/kg weight/day). The first AD injection was given two weeks after beginning the different light/darkness sessions. Ten days after the last injection, the animals were sacrificed after 24 hours of fasting.

### Sample collection and processing

Each animal was sacrificed under anaesthetic (75.0 mg ketamine/0.5 mg medetomidine/kg weight i.p.). Blood from the vascular trunk of the neck was collected in tubes to obtain blood plasma (EDTA-K_3_). The tubes were centrifuged for 15 min at 1500 g and 4°C, then the plasma was immediately collected and frozen at –85°C.

Next, and under controlled temperature conditions, the heart, kidney, brain, liver and pineal gland were extracted and weighed and the corresponding homogenates immediately made with a mechanical rotary homogenizer (Tempest Virtis). The buffer used for the homogenization was Tris-HC1 (20 mM) at a pH of 7.4.

24 hour samples of urine were collected in metabolism cages 24 hours before sacrifice. Rats were acclimatised to the metabolism cages for 48 h prior to 24 h urine collection. They were immediately centrifuged (1250 g for 10 min at 4°C) and their parameters analyzed.

### Variables analyzed

The following parameters were analyzed in all the animals studied:

  Evolution of their weight (%): at the beginning of the study (1^st^ weighing), at a fortnight before administering AD (2^nd^ weighing), and prior to sacrifice (3^rd^ weighing).
  General biochemistry: i) in plasma: urea (mg/dL), creatinine (mg/dL), uric acid (mg/dL), total proteins (g/dL), albumen (g/dL), lactate dehydrogenase (LDH;U/L), creatinine-quinase (CK;U/L), aspartate aminotransferase (AST;U/L), alanine aminotransferase (ALT;U/L) total cholesterol (mg/dL); ii) in urine: urea (mg/24h), creatinine (mg/24h), total proteins (mg/24h) and microalbumen (µg/24h). All the parameters were analyzed by spectrophotometry in the Modular Analytics autoanalyser (Roche/Hitachi Ltd Tokyo, Japan) at the Clinical Analyses service of the Reina Sofia Teaching Hospital, Córdoba.
  Biomarkers of oxidative damage in kidneys, heart, liver and brain: lipoperoxides (LPO; µmol/mg protein), total glutathione (TG; µmol/mg protein), reduced glutathione (GSH; µmol/mg protein), catalase (CAT;AU/mg protein), glutathione peroxidase (GPx;AU/mg protein), glutathione reductase (GRd;AU/mg protein) and glutathione-S transferase (GST;AU/mg protein). They were all analyzed by spectrophotometry with Bioxytech S.A. (Oxis International; Portland, OR, USA) reagents in the Department of Biochemistry and Molecular Biology at the Faculty of Medicine, Córdoba University. The analyser used was a Shimadzu (UV-1603) spectrophotometer.

For the lipid peroxidation (LPO) we used the reagent kit LPO-586, which evaluated the formation of LPO products through concentrations of malonildialdehyde (MDA) and 4-hydroxyalkenal (4-HDA). Briefly, one molecule of MDA or 4-HDA interacts with two of the chromogen N-methyl 2-phenylindole, originating a stable chromophore, which presents its maximum absorbance at 586 nm. (MDA + 4-HDA sample)_concentration_  =  ABS sample X (Standard_concentration_)/ABSstandard.

For the TG, the reagent kit GSH-420 was used. The sample was buffered and the reducing agent Tris (2-carboxyethylphosphine) added to reduce all the oxidized glutathione to a smaller size. The chromogen 4-chloro-methyl-7-trifluoromethyl-quinoline metasulphate formed thioesters with all the thiols present in the sample. Its absorbance at 420 nm was linearly proportional to the GSH concentration. A calibration curve was made by the linear regression of the absorbance against the concentration.

GSH used the reagent kit GSH-400: the reaction between the reagent 4-chloro-methyl-7-trifluoromethyl-quinoline metasulphate and all the mercaptans (R-SH) forms thioesters. Afterwards, under alkaline conditions, the reaction of elimination with the reagent 30% sodium hydroxide was produced, obtaining with GSH a chromophore group, which presented its maximum absorbance at 400 nm (GSH_concentration_)  =  ABS sample X (Standard_concentration_)/ABSstandard.

The Aebi [29] method was used for the catalase (E.C.:1.11.1.6) determination. The hydrogen peroxide, decomposed by the catalytic activity of the enzyme, showed an increase in optical density as it descended the length of the wave (λ). The hydrogen peroxide decomposition can be measured directly by the decline occurring at 240 nm for 0, 15, 30, 45 seconds with the aim of assessing the kinetic stabilization of the process. Activity =  K (1/t)×Ln (A1/A2) where A1: Absorbance at 0 seconds; A2: Absorbance at 15 seconds; t: Interval of time between A1 and A2 expressed in minutes; Ln: Napierian logarithm.

For the determination of the GPx (E.C.:1.11.1.12) and GRd (E.C.:1.6.4.2) was used the Flohe and Gunzler [30] method. The GSH-Px assay is based on the oxidation of NADPH to NAD+, catalyzed by a limiting concentration of glutathione reductase, with maximum absorbance at 340 nm. For this purpose, a volume equivalent to 25 µg of proteíns was taken from the sample,adding to it a potassium phosphate buffer of 0,1 M, at a volume necessary for obtaining 880 µL, 53 µL of glutathione-reductase, 133 µL of GSH and 100 µL of NADPH. The microcuvette was shaken inverting it and then incubated in a thermostatized bath at 37°C for three minutes. After this, the microcuvette was removed from the bath and 100 µL of tert butyl-hydroperoxide was added to the sample, shaking it again by inversion and placing it in the spectrophotometer. Next, a reading was taken at a wavelength of 340 nm for 5 minutes, measuring the decrease in the absorbance of the sample every 30 seconds. The formula applied was as follows: U, Units (µmol/min)  =  ΔABS/min/6.22 where the denominator was the coefficient of molar extinction for the NADPH, which was of 6.22 cm^2^ µMol^−1^; U  =  Enzyme units which transform 1 µmol of corresponding substrate into the time unit (minute) and ΔABS  =  Increment or decrement of absorbance. For the GRd the NADPH dissolution prepared for the GPx was employed and 100 µL of oxidized glutathione was added. The absorbance was read and calculated in the same way as for the GPx. This flavoprotein contains 1 mol of flavin adenine dinucleotide (FAD) and its main function is to maintain the intracellular concentrations of GSH.

Finally, the Warholm et al. [31] method was used for the glutathione-S transferase (GST; E.C.: 2.5.1.18), a cytosolic enzyme which catalyzes the reaction of the glutathione (acting as a nucleophil) with a wide variety of electrophilic substrates. The amounts of sample and buffer were the same as those employed in the analysis of the previous enzymatic activities. 100 µL of reduced glutathione was added and the solution agitated inverting it and then incubated for 3 min. at 37°C. Next, 50 µL of 1-chloro 2,4-Dinitrobenzene (CDNB) was added, the solution was again agitated inverting it and the spectrophotometric reading made at a wavelength of 340 nm every 30 seconds.

  Hormonal determinations: Melatonin (MEL; pineal gland tissue -mg/mg protein- and plasma -pg/mL-) and leptin (LEP; plasma -pg/mL-) were quantified by ELISA (Oxford Biomedical Research EA 65, USA) in the Nuclear Medicine service at Reina Sofia hospital, Córdoba.

### Statistical study

The statistical study was made with the SPSS (SPSS INC. Version 15 for Windows) application. First, the fit to normality of the different qualititative variables comprised in the study through the application of the Kolmogorov-Smirnov test was verified. Once it was seen that the values fitted a normal distribution, a trial based on the F of Snedecor was applied as a test of the homokedasticity of the variance. The hypothesis contrast of each of the variables in the different groups conformed was made by applying a one route analysis of variance and two factors (ANOVA), with a fit for Sidak's multiple comparisons. The minimum significance level was of 95% (p<0.05). The results were expressed as an arithmetic mean ± standard deviation (SD).

For the comparison of the variables which did not fit a normal distribution (MEL in a pineal gland tissue pool), Mann-Whitney's non parametric U test was used. 

Likewise, the “r” correlation coefficients of Pearson and of Spearman were considered to estimate the association between the most important variables of the study.

## Results

### Weight evolution

As seen in Table 1, all the groups treated with AD underwent a significant decrease in body weight compared with the respective untreated groups (*p<*0.001). This weight loss percentage was significantly lower in group AD+D. The gain in weight in the untreated groups was significantly higher in photoperiod D vs. the control.

**Table 1:** Weight percentage differences between the first weighing (at the beginning of the study), second weighing (at two weeks before initiating the administration of Adriamycin) and third weighing (before sacrifice) for each of the study groups (N = 36; 6 animals/group).

	Weight percentage	
Groups (n = 6)	Differences between the first and the second weighing (%)	Differences between the second and the third weighing (%)
**Control**	17.93 (2.41)	4.90 (4.29)
**L**	16.75 (8.92)	5.24 (3.31)
**D**	25.29 (10.39)^a^	14.06 (5.31)*^b^*
**AD**	−9.33 (5.40)^a^	−17.82 (3.27)^a^
**L+ AD**	−7.70 (3.35)	−17.81 (4.84)
**D+ AD**	−0.08 (0.07)^i^	−10.93 (5.67)

Control (12h light/12h darkness; L:D); exposure to permanent light (24h L); exposure to darkness (22h D) with light sessions between 4 and 6 p.m.; injected with adriamycin (AD), 12h/12h L:D; AD+L; AD+D. P<0.05 minimum significance level.

a *p<0.001 vs control; ^b^p<0.01 vs control.*

i *p<0.05 L+AD or D+AD vs AD.*

### Effect of the photoperiod on the melatonin and leptin levels

**Figure 1:**
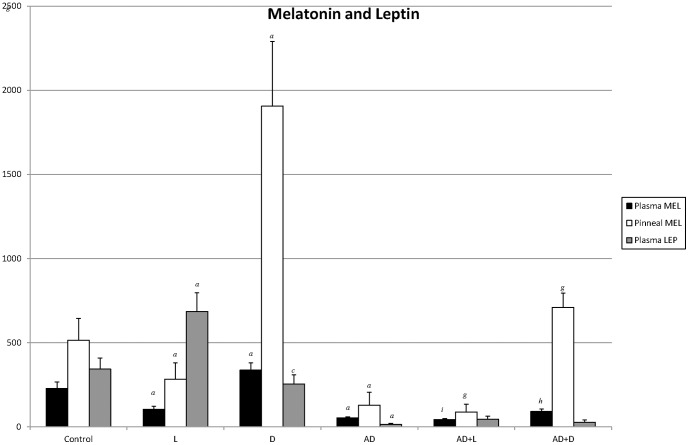
Melatonin and Leptin (mean ± SD): Hormonal determinations of melatonin (MEL) in plasma and pineal tissue and of plasma leptin (LEP) in each group (N = 36; 6 animals/group). Control 12h light/12h darkness; L:D); exposure to permanent illumination (24h L); exposure to darkness (22h D) with light sessions between 4 and 6 p.m. injected with Adriamycin (AD) (12h/12h L:D); AD + L; AD + D. P<0.05 minimum significance level. *^a^p<0,001* vs control; *^c^p<*0,05 vs control; *^g^ p<0,001* L+AD or D+AD vs AD; *^h^p<0,01* L+AD or D+AD vs AD; *^i^p<0,05* L+AD or D+AD vs AD.

Figure 1 shows that the data obtained in the healthy animal experienced a significant drop in MEL (in plasma and pineal gland tissue) in the permanent light periods (L), and a significant increase in the darkness (D). The photoperiod in the plasma levels of LEP had an opposite effect. The administration of AD produced a significant decline in MEL and LEP levels with respect to the control. The AD animals exhibited differences with the photoperiod in levels of MEL (plasma and pineal gland tissue), with higher values of this hormone in nighttime periods and lower values in daytime ones (AD+L or AD+D vs AD).

A significant negative correlation between the plasma melatonin and plasma leptin was established with a value of r^2^ = 0.9174 (LEP = −1.865MEL+844.28) for the healthy animal. However, there was no correlation between these variables in the animals treated with AD (r^2^ = 0.068).

### Changes in the general biochemical profile triggered by changes in the light/darkness cycle

  in plasma: No differences in the values obtained in healthy animals for any of the parameters between the photoperiod and the control were observed (Table 2). AD administration produced an increase in urea, creatinine, uric acid, cholesterol, LDH, CK and transaminases (ALT and AST) and a decline in total protein and albumen with respect to the control animals. Permanent light in the AD animal increased urea and creatinine levels and darkness decreased them as well as their cholesterol values. Significant differences between the light and darkness periods were established in the AD animal with significant increases in proteins (total protein and albumen) and diminutions in cholesterol in the nighttime photoperiod (AD+D vs.AD+L).
  in urine: An increase in microalbumen, total protein, urea and creatinine concentrations were observed in animals treated with AD with respect to the control. In the AD animal, light increased the values of excreted microalbumen and total protein values, whereas darkness diminished the excretion of urea in urine with respect to the control and the opposite photoperiod (Table 3).

**Table 2:** Plasma biochemical values (mean ± SD) of each of the parameters indicated in each group of animals (N = 36; 6 animals/group).

Plasma biochemical values						
Groups (n = 6)	Control	L	D	AD	AD +L	AD+ D
Urea (mg/dL)	32.21 (2.04)	33.66 (2.66)	33.83 (2.48)	56.66 (2.24)^a^	66.41 (2.44)*^h^*	44.15 (3.33)^g^
Creatinine (mg/dL)	0.41 (0.04)	0.40 (0.06)	0.38 (0.04)	1.76 (0.50)^a^	2.43 (0.49)^g^	1.22 (0.44)*^h^*
Total proteins (g/dL)	6.55 (0.37)	6.18 (0.76)	6.70 (0.21)	4.46 (0.41)^a^	4.17 (0.68)	5.44 (0.46)^k^
Uric acid (mg/dL)	0.35 (0.13)	0.50 (0,26)	0.25 (0.10)	1.08 (0.21)*^b^*	1.33 (0.38)	0.98 (0.16)
Albumen (g/dL)	4.17 (0.16)	3.67 (0.58)	4.55 (0.27)	2.90 (0.23)*^b^*	2.45 (0.38)	3.75 (0.63)*^l^*
LDH (U/L)	461.93 (95.42)	439.67 (98.13)	406.17 (58.46)	1208.00 (350.18)^a^	1529.88 (199.48)	1429.40 (144.42)
CK (U/L)	3612.58 (620.54)	4114.00 (1195.94)	3472.50 (1264.12)	8253.10 (972.37)^a^	8811.00 (602.70)	7856.40 (736.02)
ALT (U/L)	18.17 (1.60)	19.52 (3.51)	21.00 (3.90)	38.78 (6.42)^a^	43.73 (7.03)	41.80 (6.42)
AST (U/L)	110.27 (8.23)	116.85 (10.68)	107.67 (6.89)	210.70 (39.76)^a^	233.27 (32.82)	186.20 (21.51)
Cholesterol (mg/dL)	56.98 (14.94)	50.42 (10.42)^f^	59.10 (8.45)	72.72 (9.33)*^c^*	76.87 (11.94)*^l^*	56.00 (8.15)*^i^*

Control (12h light/12h darkness; L:D); exposure to permanent light (24h L); exposure to darkness (22h D) with light sessions between 4 and 6 p.m.; injected with Adriamycin (AD), 12 h/12 h L:D; AD+L; AD+D. P<0.05 minimum significance level. LDH: lactate dehydrogenase; CK: creatinine-quinase; AST: aspartate aminotransferase; ALT: alanine aminotransferase.

*^a^p<0,001* vs control; *^b^p<0,01* vs control; *^c^p<0,05* vs control;

*^f^p<0,05* L vs D.

*^g^p<0,001* AD+L or AD+D vs AD; *^h^p<0,01* AD+L or AD+D vs AD; *^i^p<0,05* AD+L or AD+D vs AD;

*^k^p<0,01* AD+L vs AD+D; *^l^p<0,05* AD+L vs AD+D.

**Table 3:** Urinary parameter values (N = 36; 6 animals/group) after the extraction of urine in metabolism cages 24 h before the animal's sacrifice.

Urinary parameter values						
Groups (n = 6)	Control	L	D	AD	AD +L	AD+ D
Microalbumen(µg/24h)	36.59 (7.56)	33.53 (4.07)	30.12 (13.43)	103.36 (21.75)^a^	203.81 (25.86)^g^^,^ j	106.75 (26.98)
Total proteins (mg/24h)	2.30 (0.90)	4.41 (2.07)	3.43 (1.03)	19.06 (3.12)^a^	28.21 (3.44)*^i, l^*	18.30 (1.82)
Urea (mg/24h)	115.21 (49.73)	119.08 (40.03)	118.38 (23.47)	434.61 (13.44)^a^	466.19 (23.39)*^l^*	370.40 (33.40)*^i^*
Creatinine (mg/24h)	2.43 (1.40)	2.61 (1.46)	2.11 (1.43)	13.54 (2.56)^a^	14.40 (1.93)	12.32 (2.99)

Control (12h light/12h darkness; L:D); exposure to permanent light (24h L); exposure to darkness (22h D) with light sessions between 4 and 6 p.m.; injected with Adriamcyn (AD), 12h/12h L:D; AD + L; AD + D. P<0.05 minimum significance level.

a *p<0,001* vs control.

g *p<0,001* AD+L or AD+D vs AD; *^i^p<0,05* AD+L or AD+D vs AD.

j *p<0,001* AD+L vs AD+D; *^l^p<0,05* AD+L vs AD+D.

### Effects of light/darkness rhythm on the oxidative damage biomarkers

**Figure 2:**
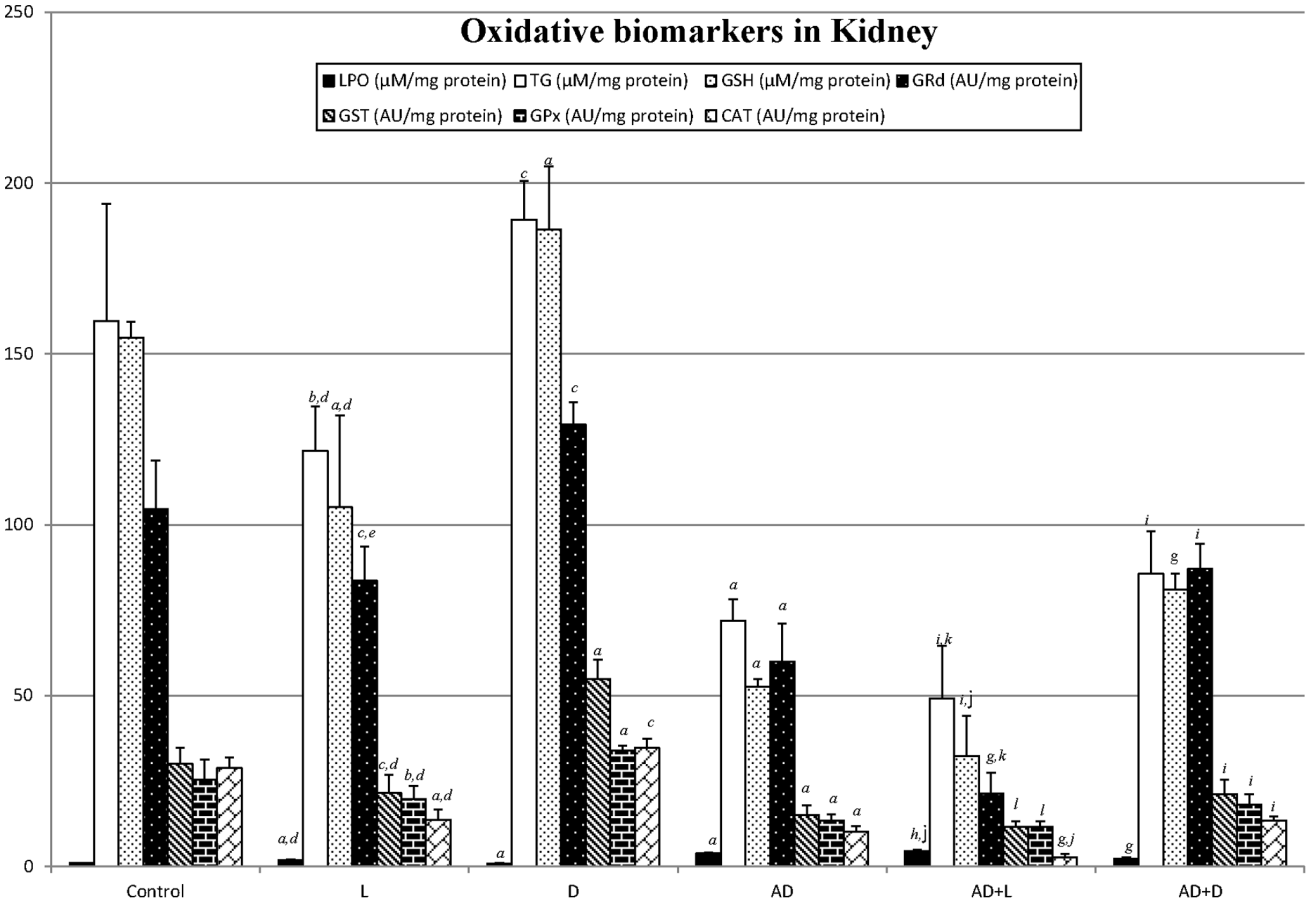
Oxidative damage biomarkers in kidney (mean ± SD): lipoperoxides (LPO), total glutathione (TG), reduced glutathione (GSH), catalase (CAT), glutathione peroxidase (GPx) glutathione reductase (GRd) and glutathione transferase (GST) for each of the groups (N = 36; 6 animals/group). Control (12h light/12h darkness; L:D); exposure to permanent illumination (24h L); exposure to darkness (22h D) with light sessions between 4 and 6 p.m.; injected with Adriamycin (AD) (12h/12h L:D); AD + L; AD + D. P<0.05 minimum significance level. *^a^p<0,001* vs control; *^b^p<0,01* vs control; *^c^p<0,05* vs control; *^d^p<0,001* L vs D; *^e^p<0,01* L vs D; *^f^p<0,05* L vs D. *^g^p<0,001* AD+L or AD+D vs AD; *^h^p<0,01* AD+L or AD+D vs AD; *^i^p<0,05* AD+L or AD+D vs AD. *^j^p<0,001* AD+L vs AD+D; *^k^p<0,01* AD+L vs AD+D; *^l^p<0,05* AD+L vs AD+D.

  Kidney tissue (Figure 2): In the animals treated with AD a significant increase in renal lipoperoxidation with respect to the healthy animal was observed. This situation was intensified in the presence of light both in healthy animals (control) and in the AD treated ones (L and AD+L).

The glutathione redox system concentration diminished in the AD animal with respect to the control and, in both AD rats and the control, light caused a more notable drop in the glutathione values whereas darkness increased them, except that light did not provoke any significant decrease in GST in the AD animal (AD+L vs. AD).

As for the antioxidant enzymes, catalase and GPx followed the same pattern of the glutathione redox system with the same exceptionality for GPx as for GST in the injected animal (AD+L vs. AD).

**Figure 3:**
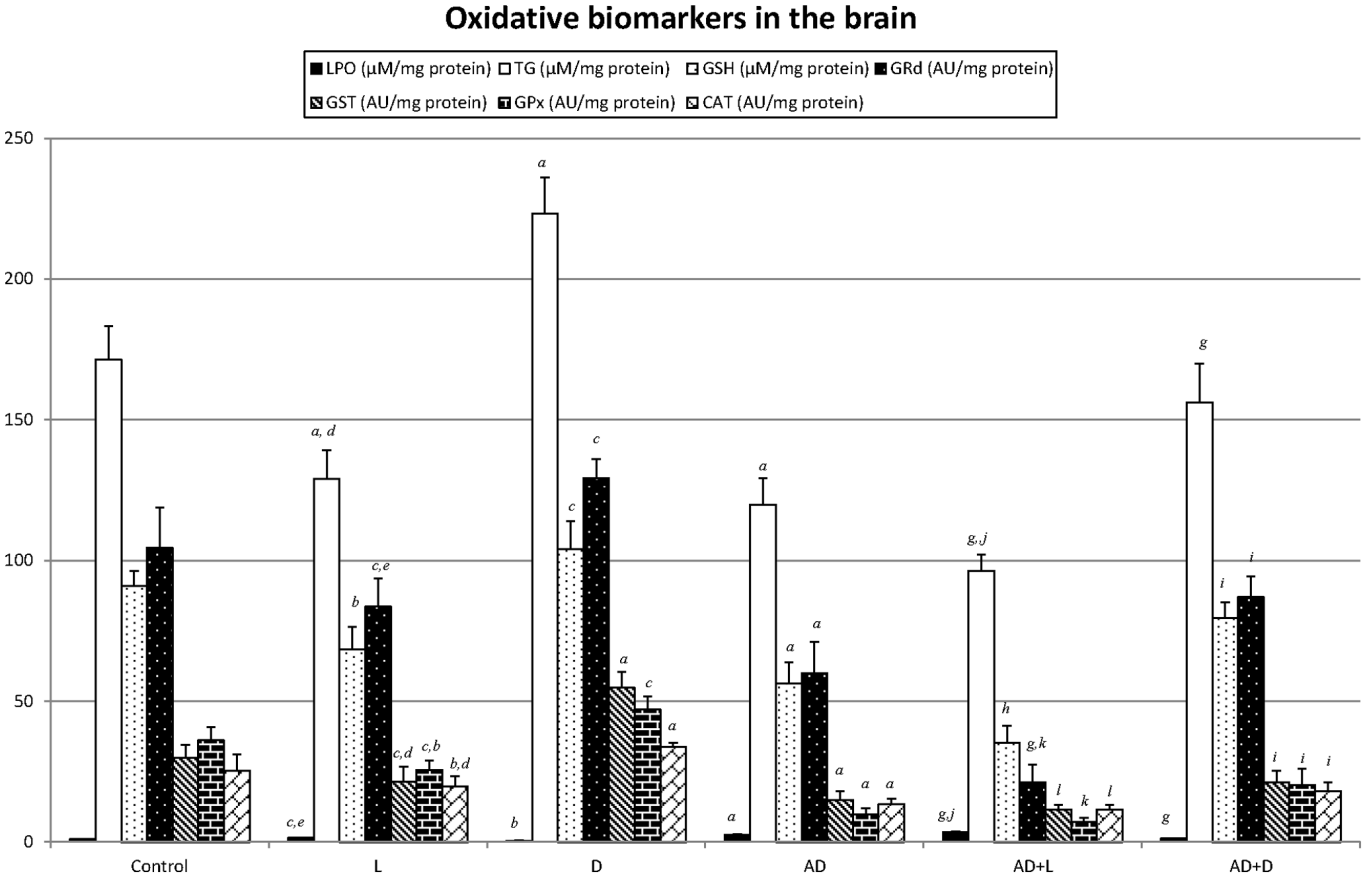
Oxidative damage biomarkers in the brain (mean ± SD): lipoperoxides (LPO), total glutathione (TG), reduced glutathione, catalase (CAT), glutathione peroxidase (GPx), glutathione reductase (GRd) and glutathione transferase (GST) for each group (N =  36; 6 animals/group). Control (12h light/12h darkness; L:D); exposure to permanent illumination (24h L); exposure to darkness (22h D) with light sessions between 4 and 6 p.m.; injected with Adriamycin (AD) (12h/12h L:D); AD +L; AD + D. P<0.05 minimum significance level. *^a^p<0,001* vs control; *^b^p<0,01* vs control; *^c^p<0,05* vs control; *^d^p<0,001* L vs D; *^e^p<0,01* L vs D; *^f^p<0,05* L vs D. *^g^p<0,001* AD+L or AD+D vs AD; *^h^p<0,01* AD+L or AD+D vs AD; *^i^p<0,05* AD+L or AD+D vs AD. *^j^p<0,001* AD+L vs AD+D; *^k^p<0,01* AD+L vs AD+D; *^l^p<0,05* AD+L vs AD+D.

**Figure 4:**
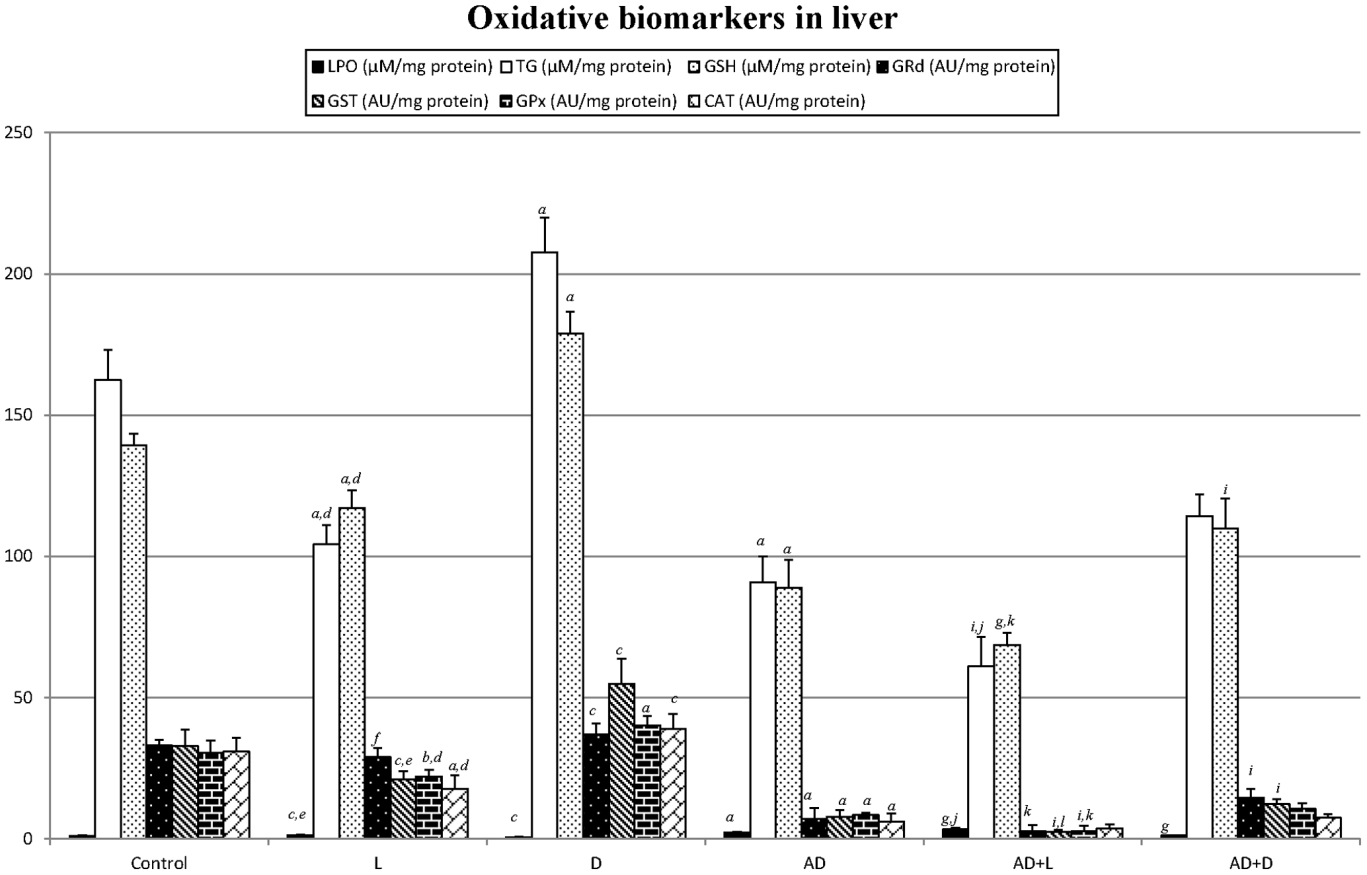
Oxidative damage biomarkers in liver (mean ± SD): lipoperoxides (LPO), total glutathione (TG) reduced glutathione (GSH), catalase (CAT), glutathione peroxidase (GPx), glutathione reductase (GRd) and glutathione transferase (GST) for each group (N = 36; 6 animals/group). Control (12h light/12h darkness; L:D); exposure to permanent light (24h L); exposure to darkness (22h D) with light sessions between 6 and 6 p.m. ; injected with Adriamycin (AD) (12h/12h L:D); AD + L; AD + D P<0.05 minimum significance level. *^a^p<0,001* vs control; *^b^p<0,01* vs control; *^c^p<0,05* vs control; *^d^p<0,001* L vs D; *^e^p<0,01* L vs D; *^f^p<0,05* L vs D. *^g^p<0,001* AD+L or AD+D vs AD; *^h^p<0,01* AD+L or AD+D vs AD; *^i^p<0,05* AD+L or AD+D vs AD. *^j^p<0,001* AD+L vs AD+D; *^k^p<0,01* AD+L vs AD+D; *^l^p<0,05* AD+L vs AD+D.

  Brain tissue (Figure 3): The brain tissue followed the same behaviour pattern as the renal one for lipoperoxidation, the glutathione redox system and the antioxidant enzymes.
  Hepatic tissue (Figure 4): In the animals treated with AD, a significant increase in their hepatic lipoperoxidation was observed with respect to the healthy animal. This situation worsened in the presence of light in both groups (L and AD+L).

The glutathione redox system concentration decreased in the AD animal with respect to the control, and in both cases light caused a more notable decline in the glutathione values (except for the GRd) whereas they increased with darkness except for the TG in the AD animal (AD+L vs. AD).

With regard to the antioxidant enzymes catalase and GPx, these decreased after AD administration with respect to the control. Light produced a significant decline in the value of the antioxidant enzymes in the healthy animal as opposed to darkness in which it increased in the concentrations of antioxidant enzymes. In the AD animal, only the GPx concentrations decreased in the presence of permanent light (AD+L vs. AD).

  Cardiac tissue (Table 4): The behaviour pattern of the cardiac tissue for the lipoperoxidation was the same as that for the hepatic, renal and cerebral tissue.

**Table 4:** Oxidative damage biomarkers in heart (mean ± SD): lipoperoxides (LPO), total glutathione (TG) reduced glutathione (GSH), catalase (CAT), glutathione peroxidase (GPx), glutathione reductase (GRd) and glutathione transferase (GST) for each group (N = 36; 6 animals/group).

Oxidative biomarkers in heart							
Groups (n = 6)	LPO (M/mg protein)	TG (M/mg protein)	GSH (M/mg protein)	GRd (AU/mg protein)	GST (AU/mg protein)	GPx (AU/mg protein)	CAT (AU/mg protein)
Control	1.66 (0.18)	458.16 (92.08)	133.03 (12.19)	11.88 (1.10)	3.00 (0.44)	7.77 (0.80)	51.39 (3.56)
L	2,23 (0.18)*^b,^* d	232.17 (42.18)^a^^,^ d	91.71 (15.43)*^b,^* d	5.39 (1.81) a ^,^ d	1.75 (0.56)^a^	3.31 (1.05)^a^^,^ d	39.69 (4.70)*^b,^* d
D	0.98 (0.09)*^b^*	607.17 (44.58)^a^	233.85 (14.02)^a^	15.56 (0.93)^a^	3.74 (0.24)*^b^*	8.89 (0.79)	70.24 (8.24)^a^
AD	3.18 (0.27)^a^	163.05 (64.28)^a^	73.00 (9.02)^a^	5.76 (1.41)^a^	1.32 (0.42)^a^	2.56 (0.65)^a^	30.86 (3.73)^a^
AD+L	4.46 (0.44)^g^^,^ j	73.76 (7.68)*^k^*	40.17 (5.54)^g^^,^ j	1.18 (0.32)^g^ ^,*k*^	0.96 (0.39)*^l^*	0.67 (0.36)*^i,l^*	27.11 (2.55)^j^
AD+D	1.83 (0.56)^g^	214.44 (51.53)	123.83 (2.55)^g^	8.78 (2.27)*^h^*	1.80 (0.15)	5.89 (2.14)*^h^*	48.52 (3.04)^g^

Control (12h light/12h darkness; L:D); exposure to permanent illumination (24h L); exposure to darkness (22h D) with light sessions between 4 and 6 p.m. ; injected with Adriamycin (AD) (12h/12h L:D); AD + L; AD + D. P<0.05 minimum significance level.

*^a^p<0,001* vs control; *^b^p<0,01* vs control.

^*d*^p<0,001 L vs D.

*^g^p<0,001* AD+L or AD+D vs AD; *^i^p<0,05* AD+L or AD+D vs AD.

*^j^p<0,001* AD+L vs AD+D; *^k^p<0,01* AD+L vs AD+D; *^l^p<0,05* AD+L vs AD+D.

The glutathione redox system concentration was reduced in the AD animal with respect to the control, and in both cases (AD and control) light triggered a more remarkable decline in the glutathione values in the healthy animal and in the GSH and GRd of the AD animal, and darkness made them increase in healthy animals and in the GSH and GRd of the AD animal.

The antioxidant enzymes diminished significantly in the AD animal with respect to the control. Permanent light caused a drop in the concentration of both enzymes in the healthy animal with respect to the control and in the AD animal for GPx. Conversely, permanent darkness exerted an increase in enzyme values in the AD animal (AD+D vs AD) and in the CAT of the healthy animal.

## Discussion

From the results obtained, it was deduced that MEL and LEP were influenced by the photoperiod, with an increase in the former in darkness periods and in the latter in illuminated ones. Gúndüz et al. [11] found statistically significant circadian variations in both MEL and LEP profiles. Their relationship was inverse, i.e. when MEL was high in the serum, LEP was comparably low in the Syrian hamster. During the daily light phase, transmission of adrenergic stimuli that initiated the biochemical transformation of serotonin to MEL was inhibited by light, while Bocquier et al. [32], in the ewe, showed that LEP was modulated by daylight independently of food intake, the amount of body fat, or gonadal activity.

It has been suggested that the photoperiod influences LEP concentrations and these circadian changes in LEP concentrations may be inversely linked to circulating MEL concentrations. However, some studies have reported contradictory results in the possible relationship between MEL and LEP secretion [14]–[16], [33]–[35]. In our study, a significant negative correlation was established between MEL and LEP in the healthy animal while it did not exist in the AD animal. Cardoso et al. [36] concluded that MEL exerts a permissive role in insulin-stimulated LEP production in rat adipocytes. These effects are caused by melatonin binding to the Gi protein-coupled MT1 membrane receptors and might be linked to its ability to potentiate insulin-induced phosphorylations of insulin receptors and Akt (Protein Kinase B). The lack of any correlation between the MEL and LEP levels in the AD animal could be due to the loss of weight occurring in the AD-treated rats, in which the levels of both hormones also significantly decreased. It is known that circulating leptin levels are strictly related to body mass [37], with no significant differences being established with the photoperiod in AD rats in their LEP levels (AD+D and AD+L vs AD), although differences did appear in MEL ones.

Weight loss is precisely a symptom of the hyperlipidemic nephropathy originated by the AD, along with all the biochemical and urinary changes described in the results.

Thus, the nephrotic syndrome induced by administration of AD is characterized by a decline in body weight and by proteinuria, albuminuria, hypoproteinemia, hypoalbuminemia and hyperlipidemia [38]–[40]. In our study, these symptoms appeared to be affected by the photoperiod, which improved some of them in the darkness phase (AD+D vs AD), and made them worse in the permanent light phase (AD+L vs AD). Túnez et al. [41] demonstrated that AD and constant light increased the triglyceride and cholesterol levels. These alterations were reverted toward normality by MEL administration, and this would seem to coincide with our results in which the darkness period with higher MEL values appears to improve the pathological symptoms of the AD-induced hyperlipidemic nephropathy.

The molecular mechanisms responsible for the pathogenesis of AD-induced renal injury involved oxidative stress. The clinical use of AD is restricted precisely because of its serious toxicity in various organs viz., heart, liver, lung, kidney, and testis [42]. Thus, AD administration caused a lipidic lipoperoxidation in renal, cerebral, hepatic and cardiac tissue, accompanied by a general reduction in the glutathione redox system and in the antioxidant enzymes. Reactive oxygen species may directly damage lipid membranes and they may also mediate in the activation of genes for some pro-inflammatory cytokines (TNF-a, IL-1, IL-6) through the stimulation of transcription factor NF-kB [40], [43].

The photoperiod had an influence on oxidative stress. Thus, light seemed to increase the lipoperoxide levels and reduce the antioxidative stress biomarkers in the healthy animals whereas the darkness periods had the opposite effect. A circadian secretion pattern has been described in some of the enzymes in this study and it has been related to the secretion rhythm of the melantonin. Thus, daily rhythms of antioxidant enzyme activity, as well as of glutathione and lipid peroxide levels, have been described in animal brain [44]–[45]. For instance, in a study made by Baydas et al. [18], LPO levels increased progressively in the darkness phase and reached a peak level at 12 midnight, whereas CAT and GPx activity showed maximum levels during the darkness phase in mouse and rat brain [18], [46]. The acrophases for the GPx activities occurred approximately 8 h after lights off, while a delay of about 2 h was detected in the acrophases for GRd activities in various brain parts [17]. GSH peaking levels were observed during the first half of the light period [45]. We did not study the daily rhythms of the antioxidant systems or of the lipoperoxides, but we did note how a change in the photoperiod contributed to a modification in oxidative stress biomarkers, which could be related to the hormone MEL. Thus, in a study by Pablos et al. [17], the exposure of chicks to constant light for 6 days eliminated their melatonin rhythm as well as the peaks in both GPx and GRd activities, and these authors suggested that the melatonin rhythm may be related to the nightime increases in enzyme activities. It has also been found that pinealectomy, which eliminates melatonin rhythm, has a supression effect on GPx activity levels [18]. MEL, the hormone of darkness and messenger of the photoperiod, is also well known to exhibit strong direct and indirect antioxidant properties [25]. MEL detoxifies the highly toxic hydroxyl radical as well as the peroxyl radical, peroxynitrite anion, nitric oxide and singlet oxygen. Additionally, melatonin stimulates a variety of antioxidative enzymes including superoxide dismutase, GPx and GRd [47]–[49].

During the light period, the activation of the oxidative stress could be a result of the increase in LEP since the latter augments the generation of reactive oxygen species (ROS) [26]–[27] and decreases plasma Paraoxonase 1, which protects plasma lipoproteins from oxidative modification by ROS activity [50]. However, contrary to our study, no circadian variations in oxidative stress-related GSH, GPx or lipidic peroxidation were observed in the liver or the kidney either in young or old rats exposed to light-dark cycles 12∶12 and 18∶6 [51]. Nevertheless, it was verified that pinealectomy, which eliminates melatonin rhythm, has a suppression effect on GPx activity levels [18], so that the decline in the MEL concentration and the increase in LEP which could be involved in the generation of the oxidative stress in the luminic phase, the same as occurred in our study. It has been proved that the melatonin secretion from the ovine pineal gland is negatively responsive to leptin during long days, whereas leptin can stimulate melatonin secretion during short days in seasonal-breeding mammals (ewes) [52].

In the AD animal, the light-darkness situation did not follow such a defined pattern for oxidative stress as the healthy animal. This was probably due to the lack of any correlation between MEL and LEP in the AD animal, and to LEP not experiencing any concentration variations in the AD animal because of an important loss of weight [37] induced by hyperlipidemic nephropathy.

To conclude, the administration of AD in rats generated typical symptoms of hyperlipidemic nephropathy accompanied by serious oxidative stress in renal, cerebral, hepatic and cardiac tissue. Additionally, in the healthy animal, but of less relevance in the AD animal (perhaps because of the intense damage already existing), permanent light increased the lipoperoxide levels and reduced those of antioxidant biomarkers, whereas darkness had the opposite effect. This effect of the photoperiod on oxidative stress could be related to the circadian rhythm of the inverse release shown by MEL and LEP, accentuating the release of MEL in the darkness phase and that of LEP in the light phase. The correlation between MEL and LEP in the healthy animal seemed to demonstrate the relationship between both variables, which was lost in the AD animal, possibly due to the damage to its tissues.
