# Correction: Impact of Light/Dark Cycle Patterns on Oxidative Stress in an Adriamycin-Induced Nephropathy Model in Rats

**DOI:** 10.1371/journal.pone.0113776

**Published:** 2014-11-17

**Authors:** The PLOS ONE Staff

The second author’s name is spelled incorrectly. The correct name is: Antonia Díaz-Moreno. The correct citation is:

Escribano BM, Díaz-Moreno A, Tasset I, Túnez I (2014) Impact of Light/Dark Cycle Patterns on Oxidative Stress in an Adriamycin-Induced Nephropathy Model in Rats. PLoS ONE 9(5): e97713. doi:10.1371/journal.pone.0097713
